# Image-Based Assessment of Growth and Signaling Changes in Cancer Cells Mediated by Direct Cell-Cell Contact

**DOI:** 10.1371/journal.pone.0006822

**Published:** 2009-08-31

**Authors:** Peter Lapan, Jing Zhang, Andrew Hill, Ying Zhang, Robert Martinez, Steven Haney

**Affiliations:** Department of Biological Technologies, Wyeth Research, Cambridge, Massachusetts, United States of America; University of Texas MD Anderson Cancer Center, United States of America

## Abstract

**Background:**

Many important biological processes are controlled through cell-cell interactions, including the colonization of metastatic tumor cells and the control of differentiation of stem cells within their niche. Despite the crucial importance of the cellular environment in regulating cellular signaling, *in vitro* methods for the study of such interactions are difficult and/or indirect.

**Methodology/Principal Findings:**

We report on the development of an image-based method for distinguishing two cell types grown in coculture. Furthermore, cells of one type that are in direct contact with cells of a second type (adjacent cells) can be analyzed separately from cells that are not within a single well. Changes are evaluated using population statistics, which are useful in detecting subtle changes across two populations. We have used this system to characterize changes in the LNCaP prostate carcinoma cell line when grown in contact with human vascular endothelial cells (HUVECs). We find that the expression and phosphorylation of WWOX is reduced in LNCaP cells when grown in direct contact with HUVECs. Reduced WWOX signaling has been associated with reduced activation or expression of JNK and p73. We find that p73 levels are also reduced in LNCaP cells grown in contact with HUVECs, but we did not observe such a change in JNK levels.

**Conclusions/Significance:**

We find that the method described is statistically robust and can be adapted to a wide variety of studies where cell function or signaling are affected by heterotypic cell-cell contact. Ironically, a potential challenge to the method is its high level of sensitivity is capable of classifying events as statistically significant (due to the high number cells evaluated individually), when the biological effect may be less clear. The methodology would be best used in conjunction with additional methods to evaluate the biological role of potentially subtle differences between populations. However, many important events, such as the establishment of a metastatic tumor, occur through rare but important changes, and methods such as we describe here can be used to identify and characterize the contribution of the environment to these changes.

## Introduction

Cancer is a complex disease that is often characterized as dysregulated growth [1]. While the root of cancer cell proliferation is the result of a loss of growth and cell cycle regulatory controls within the cancer cells themselves, changes in the way cancer cells interact with the surrounding environment are also critical to tumor development and clinical cancer [2], [3]. These include proliferative and invasive signals transmitted from stromal cells and proangiogenic signals from cancer cells to the endothelium. These interactions between cancer cells and their environment have been more difficult to characterize and target for therapeutic intervention than intrinsic changes to cancer cells, since *in vitro* models of cell-cell interactions are difficult to establish and standardize, especially at the scale necessary for drug screening. Despite these difficulties, the interaction between cancer cells and their environment have proven to be an effective strategy for treating cancer [4], and therefore increased attention to how cancer cells function as tumors is an important problem.

Methods for the *in vitro* study of cancer cell interactions with stromal and endothelial cells have been developed, such as how cancer cells induce angiogenesis and recruit macrophages [5]–[8]. Related methods allow the study of intercellular signaling through a coculture phase for inducing paracrine and heterotypic contact-dependent changes, followed by separation of the cell types for quantitation by transcriptional profiling, western blotting or related methods. The ability to detect changes in samples grown in direct coculture, mixed monoculture (through the use of inserts or related physical barriers), and standard monoculture using conditioned media allow such processes to be attributed to specific levels of interactions. While valuable, these systems carry limitations by relying on responses that must be averaged across the entire sample for each treatment, and typically involve significant processing to separate the cell types after direct coculture to generate homogenous samples for profiling or related analyses. The integration of quantitative fluorescence microscopy (High Content Screening, or HCS) into the early drug discovery process and basic biological research [9]–[11] offers methods for improving coculture studies by facilitating the direct measurement of morphology, proliferation and cellular signaling in cells grown in direct contact with different cell types.

We have developed and tested an algorithm for quantifying changes in epithelial cancer cells grown in direct contact with endothelial cells. The method identifies cell type and location to determine the proximity of endothelial cells to cancer cells, and quantitates cellular features, including cell health and the extent of activation of signaling pathways for cells adjacent to endothelial cells and compares these features to epithelial cells that are non-adjacent to endothelial cells. The process can be reversed, to characterize changes in endothelial cells that result from interactions with cancer cells. The effect of cancer cells on endothelial cells can be measured by comparing these two groups within the same sample without separating cells. We demonstrate the approach using prostate and breast cancer cells, and validate the method by demonstrating that gene expression changes identified in transcriptional profiling studies are observed in samples studied with the methods described.

## Materials and Methods

### Cells, media, reagents and culture conditions

HUVEC cells were purchased from Cambrex (Cat#: CC-2519) and maintained in EBM-2 medium (Endothelial Cell Growth Medium: CC-3162 with 2%FBS), HUVEC cells were cultured for no more than ten passages. The prostate cancer line LNCaP (ATCC, Manassas, VA) was grown in RPMI1640 supplemented with glutamate, non-essential amino acids, penicillin-streptomycin and 10% FBS (all from Invitrogen, Carlsbad, CA).

Mouse anti-CD31 (Cat#: 550389) was from BD Pharmingen (San Diego, CA), Mouse anti-CDw75 (IgM) (Cat #: ab9515-500) was from Abcam (Cambridge, MA), Rabbit anti WWOX (28–42) (Cat #: AP1008) was from Calbiochem/EMD Chemicals (Gibbstown, NJ), Rabbit anti-(pThr33)WWOX (Cat #: AP1009), was from Calbiochem/EMD Chemicals, mouse anti p73 (Cat #: 32-4200) was from ZyMed/Invitrogen, mouse anti-c-Jun (Cat #: OP55) was from Calbiochem, mouse anti-JNK1 (Cat #: MAB17761) was from R&D Systems (Minneapolis, MN).

### High content analysis

HUVEC and LNCaP cells were cultured separately in T75 flasks, trypsinized and mixed in a 1∶1 ratio in EBM2-2%FBS medium, at 5000 cells (total)/well into Poly-D-lysine-coated 96-well plates(BD: Biocoat 356640). 48 hr later, cells were washed once in PBS, fixed in 4% PFA for 10 min, washed twice with PBS, permeabilized by incubating in 1% Triton-X 100 for 3 min, and washed three times in PBS. All antibody staining were performed in PBS with 1% goat serum. The primary antibodies were added at their empirically-determined optimal dilution: anti-CDw75 at 1∶50; anti-WWOX, anti-pWWOX, anti-P73, anti-c-Jun, anti-JNK1 were all used at a dilution of 1∶100. After three PBS washes, Alexa-labeled secondary antibodies (Molecular Probes/Invitrogen) were added at a 1∶200 dilution and DAPI was added to stain nuclei, as indicated in the figure legends.

### Coculture, separation and RNA preparation for Transcription Profiling

LNCaP and HUVEC cells were expanded separately in T75 flasks. HUVECs were grown to 70% confluence, at which point 7×10^5^ LNCaP cells were seeded, and cells were grown in EBM2-2% FBS for 48 hr. Cells were trypsinized and resuspended in 1 ml (for one T75 flask) ice-cold PBS/0.1%BSA(<4×10^6^ cells/ml), 25 µl CD31-bound Dynabeads (4×10^8^ beads/ml) (Invitrogen:111-55D) were added, mixed well and incubated at +4C with gentle tilting and rotation for 20 min. 1 ml ice-cold PBS/0.1%BSA was added and the sample was placed on a Dynal MPC (Magnetic Particle Concentrator, Invitrogen) for 2 min to sediment the beads. The supernatant, which contained the LNCaP cells, were saved separately, and the beads were resuspended in 1 ml ice-cold PBS/% 0.1BSA again, and the supernatant was removed and saved. The wash process was repeated 4 times and the supernatants pooled. The pool was spun down and the pellet was labeled the LNCaP fraction and the beads were labeled as the HUVEC fraction. RNA lysis buffer was added into both fractions and RNA was purified using a Qiagen miniRNA kit (Valencia, CA). mRNA samples were prepared and processed for transcriptional profiling using the Affymetrix U133A GeneChips as described previously [12].

### Proximity algorithm

Cell cocultures are stained with DAPI, which will stain the nuclei of all cells, and a second cell type specific stain. We are using either CD44 to identify HUVEC cells or CDw75 to identify LNCaP cells. Fluorescence is quantified using a Cellomics VTi high content scanner. Images were analyzed in CellProfiler [13] provided the x-y coordinate location of a nucleus within a given image. Using the nuclear x-y coordinates, the x and y offsets of one cell from another cell can be calculated. These offsets provide the length of the sides of a right triangle.

The distance d between the two nuclei is calculated using these offsets and the Pythagorean Theorem, where
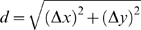
and 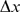 is the x distance between x_1_ and x_2_ and 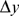 is the y distance between y_1_ and y_2_. If the distance d is less than the average diameter of the cells, the two cells are considered to be adjacent to each other. In contrast, if the distance d is greater than the average diameter of the cells, the two cells are considered to be non-adjacent. The average diameter of the cells was determined by tabulating the “MajorAxisLength” and “MinorAxisLength” values that were calculated by CellProfiler for a set of reference images.

The nuclear distance, and the CDw75 staining or CD44 staining were then used to determine which HUVEC cells were in contact with an LNCaP cell through the following process. The first step includes identifying all cells in the overall coculture population using the nuclear stain DAPI. The second step is to subdivide the population of all cells into two subpopulations, those cells that stain with CD44 (HUVEC) and those that stain with CDw75 (LNCaP). Finally, the third step is to further subdivide the epithelial population into two subpopulations, those LNCaP cells adjacent to a HUVEC cell and those non-adjacent to a HUVEC cell. A list of all LNCaP cells (CDw75 positive or CD44 negative) and HUVEC cells (CDw75 negative or CD44 positive) was constructed. The Pythagorean distance for each LNCaP cell compared to each HUVEC cell was calculated. The distances smaller than the average diameter of an LNCaP cell were flagged as LNCaP cells adjacent to HUVEC cells.

### KS statistic

Adjacent and non-adjacent subpopulations of cells were identified using the previously described proximity algorithm. The intensity of an antigen of interest was compared across these two populations using the ks.test function in the R programming language (www.r-project.org **).** A two-sided ks.test p-value of less than 0.05 was used to infer that the two subpopulation distributions did not come from the same parent distribution.

### Mosaic analysis of adjacent and non-adjacent LNCaP cells

For the data shown in the figure, two wells were considered. From these two wells, 3428 LNCaP cells were available, and categorized as either adjacent (655) or non-adjacent (2773). From these cells, all possible 655 adjacent cells were selected, and 1000 non-adjacent cells were randomly sampled. To make a square mosaic, the first 625 cells from each of these samples were displayed in a 25×25 matrix of images. Each cell image was a 10×10 pixel square, centered on the (x,y) coordinates of a cell. Channel 3 images (p73) were displayed using the default hsv color map, with fixed 12-bit color limits (intensities from 0–4096) so that intensity scales were the same for all images.

## Results

### Development of an image-based method for the study of endothelial cells in direct contact with cancer cells

Through indirect immunofluorescence of cell type-specific antigens, we have been able to identify conditions that allow the cells of different origins to be labeled for HCS. Conditions for specifically labeling HUVEC cells have already been described, through the use of antibodies specific for PECAM, or CD31 [14]. Such labeling is used for flow cytometry-based studies and as such there was a high likelihood that such detection would be possible in an adherent culture system. Antibodies were obtained from commercial sources and with minimal screening fixation and staining conditions were determined. A wide variety of candidate markers are available for the detection of prostate carcinoma cells, most notably prostate-specific antigen (PSA) and prostate-specific membrane antigen, which are both clinically relevant to prostate cancer (although PSA itself is a secreted protein, its production can be detected within prostate cancer cells) [15]. Additional candidate markers include tumor antigens that normally show highly restricted expression (typically to fetal or limited adult tissue types). One example from this latter category is CDw75, a cell-surface marker for B cell lymphocytes produced by a beta-galactoside alpha-2,6-sialyl-transferase [16]. Expression of CDw75 antigens have been observed in many epithelial cancers, such as the stomach, and colon [17], [18]. In a screen of candidate markers for the detection of prostate carcinoma cells in coculture with endothelial cells, we found that CDw75 was a specific marker for all three prostate cell lines under study. Coculture experiments showed that the cell types could be readily and definitively identified using antibodies to these markers.

Following the identification of specific cell types in coculture, we sought to identify cells that are in heterotypic contact. Our approach to doing this was to identify all cells by type and location, and then develop a method for parsing cells in groups of adjacent and non-adjacent cells with respect the cells there target population is in coculture with. HCS is highly multiparametric, capturing between a dozen and more than 100 features per cell [10], [11], [19]. Fundamental features include DNA content, size and shape of the nucleus. Additional features are dependent on the methods used to label the cells, and can include measurements of the cytoskeleton, organelles, or specific proteins and their localization and phosphorylation status [20]. Within the context of the current study, we have developed conditions for detecting individual cells and their expression of either CD31 or CDw75 antigens, and their location in the field. The last feature allows us to determine the proximity of two cells to each other. The identification of a cell as adjacent can be strictly defined by the distance of the nuclei of a cancer cell to an endothelial cell. As such, cells can be segregated into adjacent and non-adjacent, and differences between these two groups can be determined.

**Figure 1:**
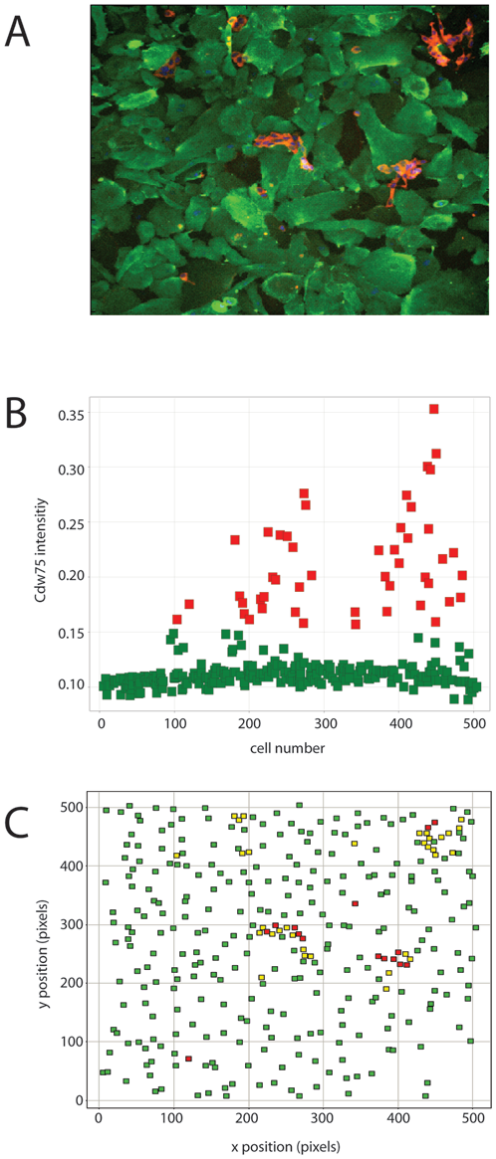
Identification of adjacent and non-adjacent endothelial cells in culture with prostate carcinoma cells. Coculture of LNCaP cells and HUVECs, LNCaP cells are labeled with anti-CDw75 antibodies and shown in red, HUVECs are labeled with anti-CD44 antibodies, in green. A. An example field image. B. Quantitation of CDw75 fluorescence used in characterizing cells as either LNCaP (high intensity, red) or HUVEC (low intensity, green). C. Mapping LNCaP cells and HUVECs. Locations can be compared to cells in panel A. Cells are identified as adjacent LNCaP in yellow, non-adjacent LNCaP cells in red and HUVECs in green.

The algorithm has been used to identify adjacent and non-adjacent LNCaP cells, relative to HUVEC cells, as shown in **Figure 1**. In **Figure 1A**, LNCaP cells, labeled with an anti-CDw75 antibody in red, are seeded with HUVECs, stained with an anti-CD44 antibody in green. The intensity of the CDw75-based fluorescence is recorded for each cell in the field. Each cell is numbered, and the intensity for each cell is shown in **Figure 1B**. From this measurement, cells can be categorized as either cancer or endothelial cells. In addition to the CDw75 staining, other properties associated with cancer cells can be used at this stage, including DNA content (many cancer cell lines, including LNCaP are aneuploid and have significantly higher DNA content than primary cells). These data can be included with the antigen intensity to score cells as cancerous or not, or in some cases can be used as a single parameter for this determination (data not shown). Once the cells have been identified, their spatial information is used to map each cell to its location. Having identified each cell as LNCaP or HUVEC, each cell is then scored for the cells that are near it, the boundary distance being set by the average diameter of the cells. Cells of one type, LNCaP in this example, are therefore further defined as adjacent to HUVEC or not. Cells identified as adjacent are shown schematically in **Figure 1C** in yellow, or non-adjacent, in red. HUVEC cells are shown as green. From this point forward, quantitative data may be extracted and analyzed for the two subsets of LNCaP cells. Differences between these subsets are indicative of a response to direct contact with HUVEC cells.

A major challenge to developing a proximity algorithm for HUVECs can be seen in **Figure 1C**. The heterogeneity in both size and shape of the cells makes it difficult to assign a distance between two cells based on the location of the nucleus. Assigning a distance from a nucleus that represents an average radius for the cell body misrepresents the interactions between many cells. Cells that vary in size, or are significantly elongated, cannot be addressed in most current image analysis algorithms. Two practical questions are (a) what steps can be instituted to minimize the effects of variability in cell size, and (b) how much error can the algorithm tolerate before an effect can not be observed? The effects of changing the way in which cells are plated was examined next, the overall impact of the variability of coculture plating conditions on the robustness of the approach in general was evaluated in the analysis section, later in this study.

### Candidate genes in endothelial cells that show changes in expression levels when cultured with cancer cells

Several studies have been published that describe changes in gene expression levels in cancer cell lines grown in coculture [21]–[25]. Each system has identified specific signaling pathway events, in both cancer and stromal cells, that are triggered by cell-cell contact. We sought to identify changes in the cell lines we are using in these studies as the most robust source of candidate proteins to be used to validate the system we have described. We used seeded LNCaP prostate carcinoma cells and HUVEC endothelial cells as the coculture system to develop a list of candidate genes for validation studies. This was accomplished using traditional methods for direct coculture, the growth of cells for a specific period of time, followed by a mechanical separation of the two cell types. Specifically, we used CD31-conjugated beads to rapidly and quantitatively separate the endothelial cells from the prostate carcinoma cells, following trypsinization of the mixed culture. The ability of the beads to separate the two cell types was characterized by immunofluorescence and by RT-PCR. The two samples of cells were checked by indirect immunofluorescence using CD31 antibodies, and it was shown that the cells separated by the beads were indeed CD31 positive, whereas the cells not bound by the beads were CD31 negative. These results indicated that the method was able to quantitatively separate the two cell types following coculture.

**Figure 2:**
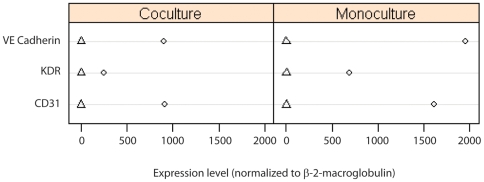
Separation of endothelial and prostate carcinoma cells after coculture. RT-PCR analysis of entothelial genes in samples of mono and coculture following a separation of the cell types. Cells grown as monocultures, either endothelial or prostate cells, were treated in a manner identical to that used to separate cells after coculture. Cells recovered by binding to ant-CD31-conjugated beads and pelleted. Expression data for each gene/condition is reported as its expression level relative to the control gene (β-2-macroglobulin) for each sample. Circles: pellet; Triangles: supernatant [depending on the Journal, they may want this info in a legend; The original figure has a legend]

Transcriptional changes that occur in LNCaP cells after coculture with HUVEC cells were identified using oligonucleotide arrays, comparing growth of the cells in coculture with HUVEC cells and after growth as a mock coculture; monoculture cells were treated with the bead-based separation protocol in a manner identical to the actual coculture samples. Prior to full transcriptional profiling by oligonucleotide microarrays, we checked the separation of the cell types by RT-PCR of three genes expressed in endothelial cells. The data are presented in **Figure 2**. Here, results for three genes are shown for the coculture and mock samples. The three genes, PECAM/CD31, KDR/MET and VE Cadherin are expressed well in HUVEC cells, but poorly in LNCaP cells, as shown in the monoculture (mock) experiments in **Figure 2**. Data from the coculture samples shows the elimination of HUVEC cells from the LNCaP cells in the supernatants to be essentially complete. Therefore, we have been able to culture LNCaP cells with HUVEC cells, and then separate the cell types for transcriptional profiling analysis.

Transcriptional profiling identified 44 genes that were upregulated by coculture with HUVEC cells, and 4 genes that were downregulated, shown in **Table 1**. Four genes were identified by two qualifiers each on the oligonucleotide arrays, MOBK1B, SASH1, TGOLN2 and WIPI49; redundant qualifiers were removed from the list. From the candidate genes on this list, we screened commercial suppliers for antibodies that would specifically identify the targets by Western blotting and indirect immunofluorescence. In many cases, commercial antibodies were not sufficiently specific, as determined by both Western blotting and HCS. Generally, the antibodies either gave a significant cross-reacting band in Western blots or did not show a titratable staining in a cellular pattern expected for the antigen. This was the case for TNFRSF19, which had the most dramatic change in mRNA expression levels. However, in the case of WWOX, identified as down-regulated by cell-cell contact in LnCaP cells, we were able to identify and validate appropriate reagents. WWOX has been well-characterized as a tumor suppressor, following studies that its expression is frequently lost as a result of translocations within the fragile site FRA16D [26], [27], including prostate cancers [28]. Although expression is lost in many cancers, its expression can frequently be observed in many cancer cell lines [29]. WWOX is phosphorylated at the Thr-33 residue in response to treatment with TNF-α and hyaluranidase [30]. Phosphorylated WWOX will then activate p53, but this induction of apoptosis is suppressed by JNK1, in part through a direct association with WWOX [31]. As such, reduced WWOX expression would also attenuate apoptosis resulting from exposure to foreign environments, such as pre-metastatic sites prior to the establishment of new tumor growth. The identification of significantly reduced expression of WWOX in LNCaP cells by contact with HUVECs presents a biologically-relevant process that could be analyzed in the system we describe.

**Table 1:** Gene expression changes of LNCaP cells grown in coculture with HUVEC.

Gene Symbol	Gene Name	Fold Change	p-Value
MYL9	myosin, light polypeptide 9, regulatory	4.68	4.84E-02
GLRX	glutaredoxin (thioltransferase)	4.07	4.94E-02
STK17A	serine/threonine kinase 17a (apoptosis-inducing)	4	5.02E-03
IL6ST	interleukin 6 signal transducer (gp130, oncostatin M receptor)	3.77	3.75E-02
MBNL2	muscleblind-like 2 (Drosophila)	3.61	1.14E-02
MGC14376	hypothetical protein MGC14376	3.59	2.92E-02
FLJ35155	hypothetical protein FLJ35155	3.56	9.32E-03
IFITM3	interferon induced transmembrane protein 3 (1-8U)	3.55	4.65E-02
OPTN	optineurin	3.25	4.00E-02
SOD2	superoxide dismutase 2, mitochondrial	3.22	4.08E-02
HES1	hairy and enhancer of split 1, (Drosophila)	3.22	4.42E-02
PTPRK	protein tyrosine phosphatase, receptor type, K	3.06	2.77E-02
CORO1C	coronin, actin binding protein, 1C	2.86	4.12E-02
ITGB5	integrin, beta 5	2.77	4.25E-02
MAN2A1	mannosidase, alpha, class 2A, member 1	2.63	3.35E-02
AOF2	amine oxidase (flavin containing) domain 2	2.61	4.90E-02
na	CDNA FLJ42565 fis, clone BRACE3007472	2.56	8.37E-03
TNFRSF21	tumor necrosis factor receptor superfamily, member 21	2.56	1.46E-02
INHBC	inhibin, beta C	2.56	2.16E-02
MGLL	monoglyceride lipase	2.52	6.08E-03
SASH1	SAM and SH3 domain containing 1	2.51	1.31E-02
MOBK1B	MOB1, Mps One Binder kinase activator-like 1B (yeast)	2.48	4.82E-02
KPNA3	karyopherin alpha 3 (importin alpha 4)	2.47	3.49E-02
RALB	v-ral simian leukemia viral oncogene homolog B (ras related; GTP binding protein)	2.44	4.80E-02
	CDNA: FLJ21778 fis, clone HEP00201	2.43	1.83E-02
APP	amyloid beta (A4) precursor protein (protease nexin-II, Alzheimer disease)	2.42	2.85E-02
(PYGO2, SHC1)	(SHC (Src homology 2 domain containing) transforming protein 1, pygopus 2)	2.4	2.85E-02
WIPI49	WD40 repeat protein Interacting with phosphoInositides of 49kDa	2.37	2.31E-02
USP48	ubiquitin specific protease 48	2.35	2.01E-03
TANK	TRAF family member-associated NFKB activator	2.33	1.53E-02
RAP2B	RAP2B, member of RAS oncogene family	2.33	3.92E-02
WDR1	WD repeat domain 1	2.32	3.45E-02
RAB3B	RAB3B, member RAS oncogene family	2.31	1.87E-02
DRCTNNB1A		2.31	2.74E-02
EHD4	EH-domain containing 4	2.29	2.64E-02
MOBK1B	MOB1, Mps One Binder kinase activator-like 1B (yeast)	2.27	2.73E-03
TAGLN2	transgelin 2	2.26	3.67E-02
TJP2	tight junction protein 2 (zona occludens 2)	2.24	4.54E-02
LRRC8	leucine rich repeat containing 8	2.22	1.40E-02
GPSM3	G-protein signalling modulator 3 (AGS3-like, C. elegans)	2.15	4.52E-02
FLJ39370	hypothetical protein FLJ39370	2.12	3.11E-02
ADSS	adenylosuccinate synthase	2.11	2.37E-02
ADAM19	a disintegrin and metalloproteinase domain 19 (meltrin beta)	2.07	3.96E-02
TGOLN2	trans-golgi network protein 2	2.02	4.67E-02
VMP1	likely ortholog of rat vacuole membrane protein 1	2	1.50E-02
			
WWOX	WW domain containing oxidoreductase	−2.02	3.61E-02
ZC3HDC6	zinc finger CCCH type domain containing 6	−2.37	4.65E-02
	CDNA FLJ40165 fis, clone TESTI2015962	−7.04	1.80E-02
TNRC9	trinucleotide repeat containing 9	−7.72	4.41E-02
TNFRSF19	tumor necrosis factor receptor superfamily, member 19	−13.24	1.88E-02

### Detection of changes in signal transduction pathways in prostate carcinoma cells resulting from cell-cell contact

**Figure 3:**
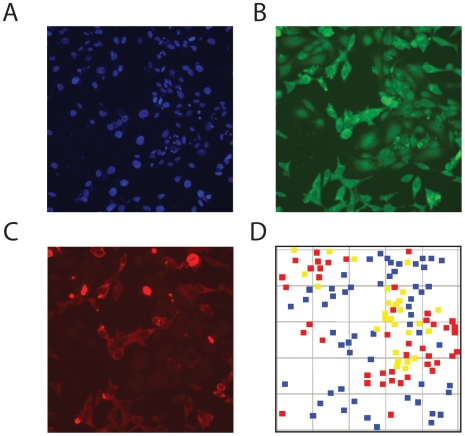
Identification of WWOX levels in prostate carcinoma cells. LNCaP cells and HUVECs in coculture were labeled using (panel A) DAPI, to identify nuclei, (panel B) anti-CDw75 antibodies, to identify LNCaP cells, and (panel C) anti-WWOX antibodies. In panel D, cells classified as HUVEC are shown as red squares, adjacent LNCaP cells as yellow squares and non-adjacent LNCaP cells as blue squares.

To test the response of WWOX levels to coculture, the methodology needed to be altered. Specifically, the method is currently not robust in four channels, due to spectral overlap in the blue to orange range of the spectra and some weakness in the intensity in the red range. As such, labeling of one antigen needed to be omitted to include WWOX. We tried omitting the CD31 antigen and testing whether CDw75 staining was sufficient to differentiate between LNCaP cells and HUVECs. Monocultures and cocultures were evaluated to determine the distribution of CDw75 staining levels in each cell type and to determine the point at which HUVECs began to be called as LNCaPs. The range of levels for the two cell types was similar to what was observed in the initial phase of the study (**Figure 1**), where LNCaP cells showed a broad range of staining, but only a few cells per field were stained lightly enough that they could be classified as HUVECs, and therefore this method of distinguishing the two cell types appeared robust enough to allow for staining of WWOX or phospho-WWOX as well. The identification of cell types and quantitation of WWOX levels are shown in **Figure 3**. Association of the signaling intensities with the correct cell type was improved over initial studies (described above) by limiting the number of cells plated, particularly for the HUVECs.

**Figure 4:**
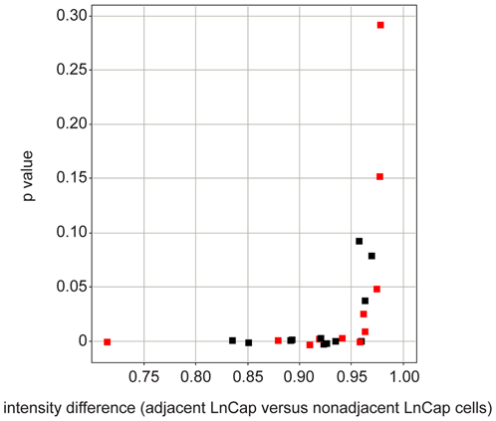
Effect of direct contact with HUVEC cells on the expression and phosphorylation of WWOX in LNCaP cells. Independent determinations, over multiple wells, of the ratio of WWOX (black squares) and phospho-WWOX (red squares) levels in LNCaP cells that are adjacent or non-adjacent to HUVECs are shown. Each point represents an individual well, where ∼350 adjacent and ∼1500 non-adjacent LNCaP cells were evaluated.

To test the image-based approach we have developed, we examined whether the changes in WWOX expression we identified in the transcriptional profiling studies could be recapitulated in LNCaP cells in the system we have described above. WWOX protein levels in LNCaP cells in adjacent and non-adjacent cells were compared. Multiple wells were used to culture LNCaP cells and HUVECs and were subsequently fixed and stained with DAPI and antibodies to CDw75 and either WWOX or phospho-WWOX. LNCaP cells were determined to be either adjacent or non-adjacent to HUVECs and analyzed for target protein levels. Average target protein levels and standard deviations were determined. From these data, the ratio and p-value of target protein levels in adjacent and non-adjacent LNCaP cells were calculated. Data from these comparisons are presented in **Figure 4**. The data shows that adjacent LNCaP cells frequently have reduced levels of both WWOX and phospho-WWOX and as the difference becomes larger, the greater the significance of the measurement. Conversely, wells not showing a difference were not as statistically robust. This seems to show that it is not always possible to make a determination about whether direct contact has had an effect, but when we can make a determination, the results show that these antigens are lower in the LNCaP cells that contact HUVEC. Wells that fail to show an effect have been compared to those that do, and we observe that wells that fail to show a difference do so as a result of too few LNCaP cells being scored as adjacent, typically through an uneven distribution of cells during plating.

**Figure 5:**
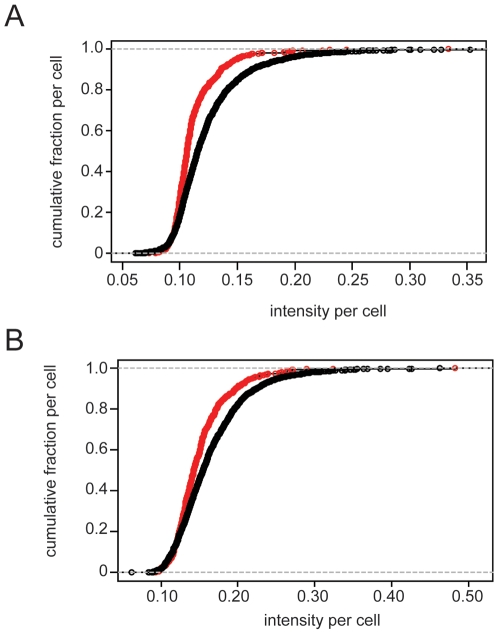
Quantitation of WWOX and phospho-WWOX levels in LNCaP cells. The amount of WWOX protein (in panel A) and phospho-WWOX protein (in panel B), are shown for LNCaP cells adjacent to HUVECs (in red) and those cells non-adjacent to HUVECs (in black). Data points represent (phospho-) protein levels per cell as a they contribute to the total antigen intensity for the entire field.

A separate observation is that although the differences in many wells are statistically significant (with p values below 1×10^−3^), the magnitude of the effect appears modest. At this stage we considered a different numerical test of the difference between target protein levels in LNCaP populations. The test we used was the Kolmogorov-Smirnov (or KS) statistic. The test compares two populations as fractional contributions to the total amount of antigen. Results for WWOX levels are shown in **Figure 5A** and for phosphorylated WWOX, which is mediated by Src, as shown in **Figure 5B**. The difference in the two curves shows that LNCaP cells adjacent to HUVECs have less WWOX protein than LNCaP cells that are non-adjacent to HUVECs when compared in a ranked listing.

Phosphorylation of WWOX by Src results in binding to JNK1, p53 and p73 [31]-[33], and these associations have been associated with reduced apoptosis. Reducing apoptosis is an essential step in tumorigenesis [2], and would be expected for cancer cells encountering non-native cell types during invasion and metastasis, and in fact this is linked directly to JNK1 function [34]. Since WWOX and pWWOX levels are reduced in adjacent LNCaP cells, we decided to examine the levels of c-Jun, JNK1 and p73 as well. Results for these proteins are shown in **Table 2**. The three proteins show varying extents of sensitivity to the proximity of HUVECs. c-Jun and JNK1 exhibit only modest effects as shown by most tests showing high p-values and ratios near 1. p73 shows a stronger effect, comparable to what was observed for WWOX and phospho-WWOX in the previous assays. This last result is consistent with findings by others, and the analysis of c-Jun and JNK1 do not refute the published studies, again suggesting that this approach is not always capable of making a determination regarding the effect of cell-cell contact, but when a determination can be made, it has supported a model of contact reducing expression and signaling of WWOX in LNCaP cells.

**Table 2:** Ratios of c-Jun, JNK1, and p73 in adjacent and non-adjacent LNCaP cells.

Protein	mean.lncap	sd.lncap	mean.adjlncap	sd.adjlncap	p-value*	ratio
c-Jun	0.0627	0.0159	0.0624	0.0205	0.4821	0.9952
	0.0636	0.0179	0.0621	0.0144	0.3239	0.9764
	0.0635	0.0213	0.0607	0.0146	0.0108	0.9559
	0.0622	0.0212	0.0594	0.0113	0.006	0.955
	0.0637	0.0161	0.0601	0.0131	6.00E-04	0.9435
	0.0694	0.0207	0.0647	0.0162	5.00E-04	0.9323
JNK1	0.0923	0.0233	0.0892	0.0222	0.033	0.9664
	0.0918	0.0242	0.0871	0.0238	0	0.9488
	0.0984	0.0285	0.0929	0.0252	2.00E-04	0.9441
	0.1004	0.0308	0.0936	0.0236	6.00E-04	0.9323
	0.091	0.022	0.0848	0.0186	0	0.9319
	0.0936	0.0353	0.087	0.0215	0	0.9295
p73	0.144	0.0439	0.1339	0.0459	0	0.9299
	0.1696	0.0689	0.1632	0.0806	0.0069	0.9623
	0.1432	0.0428	0.1311	0.0437	0	0.9155
	0.1387	0.0438	0.1206	0.0431	0	0.8695
	0.1824	0.0843	0.157	0.064	0	0.8607
	0.1851	0.0866	0.1579	0.0599	0	0.8531

Data are for each protein as determined by six independent wells each.

* p-value from T-test comparison of adjacent and non-adjacent LNCaP cells.

**Figure 6:**
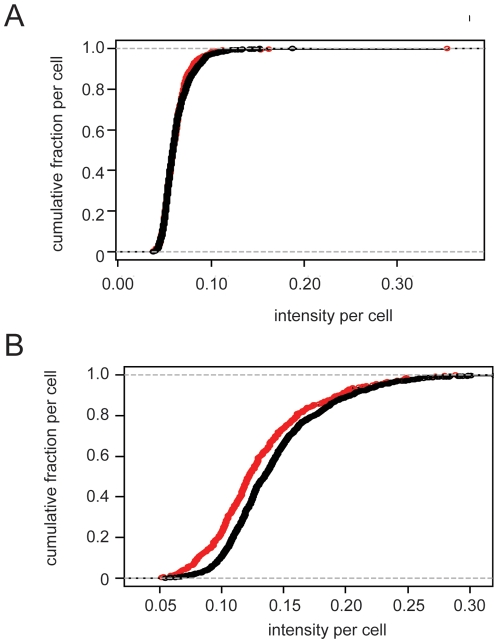
Distribution of c-Jun and p73 levels in LNCaP cells cocultured with HUVECs. Cumulative plots of c-Jun (panel A) and p73 (panel B) in adjacent and non-adjacent LNCaP cells (red and black, respectively). Data are from single wells, the ratio of c-Jun expression for the well analyzed in this figure was 0.9952, and that of p73 expression was 0.9155.

The data from these experiments were also evaluated at the population level through the KS statistic. The results for c-Jun shows that the small difference observed in the adjacent/non-adjacent ratio in **Table 2** (0.9952) are reflected in **Figure 6A** as highly coincident curves. While some of tests of coculture on c-Jun levels appear to show a difference (ratios<0.95 and a p value of 0.482), the effect is difficult to establish through multiple testing. In the case of p73, shown in **Figure 6B**, the difference of 0.9155 (p value<1×10^−3^) in the overall ratio is seen in the separation of the distribution curves. While subtle, the difference is reflected in the entire population rather than a specific subset of cells, such as a group of apoptotic cells that are specific to the adjacent cells. Statistical summaries for the comparisons in **Figure 5** and **Figure 6** are shown in **Table 3**.

**Table 3:** KS statistics of cumulative responses compared in Figures 5 and 6.

Figure	Antigen	D	kp	p
5a	WWOX	0.2543	0	0
5b	pWWOX	0.1704	0	0
6a	c-Jun	0.0495	0.4236	0.4821
6b	p73	0.1579	0	0

**D**: the KS value (maximum difference along the y-axis).

**kp**: probability that the KS differences seen would be seen by chance alone.

**p**: t-test for different means.

**Figure 7:**
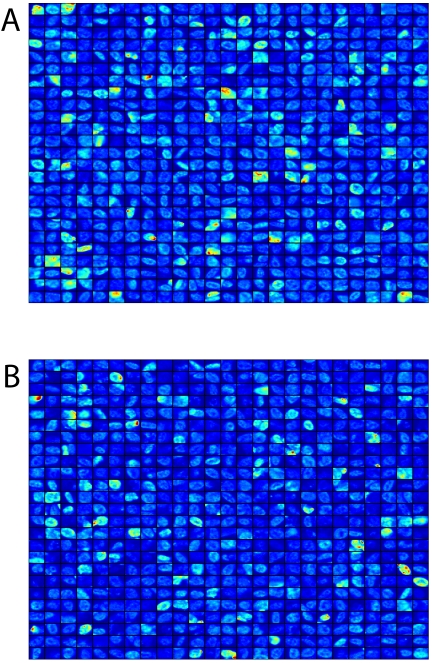
Image mosaic analysis of p73 levels in prostate carcinoma cells. Antigen intensities in LNCaP cells are clustered by proximity to HUVECs to allow overall comparisons of aggregate intensity levels between groups. Top: LNCaP cells adjacent to HUVECs; bottom: LNCaP cells non-adjacent to HUVECs.

As noted previously, one of the major challenges to this approach is the subtly of the measurements. Specifically, changes in two groups of cells are detected using population-based methods, such as the KS statistic, and need to be reconciled with direct observation wherever possible. This is difficult to achieve by examining single fields of cells, where the variability in each sample group can make group-wide trends difficult to detect. To address this, we have grouped images of cells into mosaics, which provide a stronger visual effect based on larger groups of cells within each class. The results for the p73 in adjacent and non-adjacent LNCaP cells are shown in **Figure 7**. The mosaics support the analytical methods identification of p73 levels being lower inLNCaP cells that contact HUVECs.

## Discussion

The interactions between different cell types have long been recognized as important to basic biology and therapeutic function, but methods for their study have been difficult to establish. *in vitro* biology requires reproducibility and quantitation, and drug discovery requires these factors to a greater extent. The heterogeneity of primary cells in culture, and of the responses observed in complex systems in general, have been barriers to their study. This is changing with the advent of quantitative microscopy and informatic approaches to large datasets [35], [36]. These studies have established the use of morphological and multiparametric methods for the study of genetic and chemical perturbations on cells in coculture.

The data described here presents a general method for measuring changes in cultured cells grown in direct contact with cells of another type. The methodology has been validated using HUVEC and human cancer cell lines, but could be readily extended to any two cell types that can be distinguished morphologically or immunofluorescently, including macrophages, pericytes, adipocytes, neurons or stem cells.

The method is quite flexible. This methodology can be applied to tissue samples [37], enabling comparisons between *in vitro* and *in vivo* samples for a given cell type and treatment. We have also been able to classify cancer and normal cells solely on DAPI staining intensity. Many cancer cell types are aneuploid. Depending on the extent of aneuploidy, cancer cells can be distinguished from diploid endothelial by their increased DNA content and staining with DAPI. A direct comparison of the staining intensity of cancer and endothelial cell nuclei can identify a staining threshold that can be used to sort a coculture into its two constitutive cell types. Once the two separate cell types have been identified, further subdivision of the endothelial population into adjacent and non-adjacent cells proceeds as described previously.

The advantages of this method over others, such as transcriptional profiling, are (a) significantly fewer manipulations are needed to prepare samples between culturing and fixation or lysing, which can affect some of the responses to a perturbation, (b) effects from direct cell-cell contact can be distinguished from secretion-mediated signaling, and (c), single cell events are recorded, allowing for powerful statistical tests to used in evaluating samples. This is not to say that other methods are incapable of detecting important signaling events between cells. Indeed, we have combined transcriptional profiling and HCS to characterize the effect of cell-cell contact on WWOX-JNK signaling.

The utility of methods for the study of signaling events mediated by cell-cell contact is becoming better appreciated. Probably the best recognized area of research that takes tissue organization and cell-cell contact into consideration is stem cell biology. The growth and differentiation of stem cells is explicitly controlled by their interactions with other cell types within the niche, and exclusion of a pluripotent stem cell from the niche begins the cascade of steps towards one or more differentiated cell types [38], [39]. Some of these concepts have been incorporated into the model of cancer stem cells, or tumor-initiating cells, and their role in tumor progression and chemotherapeutic resistance [40]. While some aspects are controversial (such as whether cancer stem cells originate from normal stem cells, or merely re-express a few stem cell markers), one of the principal aspects of the model is that these cells reside in hypoxic regions of tumors and produce rapidly proliferating cells that migrate to peripheral regions of the tumor and to distant sites in the body. The methods described in this report have been developed to provide better data on cell-cell interactions for any system where the events can be detected fluorescently or through morphological changes in one or more cell type where the cell types can be distinguished, including systems described above, where the context of the cell is an important factor in its growth and signaling.
